# Transcriptional pathways associated with the slow growth phenotype of transformed *Anaplasma marginale*

**DOI:** 10.1186/1471-2164-14-272

**Published:** 2013-04-22

**Authors:** Sebastián Aguilar Pierlé, Gena Kenitra Hammac, Guy H Palmer, Kelly A Brayton

**Affiliations:** 1 Program in Genomics, Department of Veterinary Microbiology and Pathology, Paul G. Allen School for Global Animal Health, Washington State University, Pullman, WA, 99164-7040, USA

**Keywords:** Intracellular bacteria, Slow growth phenotype, Global transcription, Pathway analysis, RNA-seq, *Rickettsiales*, *Anaplasma*

## Abstract

**Background:**

The ability to genetically manipulate bacteria has been fundamentally important for both basic biological discovery and translational research to develop new vaccines and antibiotics. Experimental alteration of the genetic content of prokaryotic pathogens has revealed both expected functional relationships and unexpected phenotypic consequences. Slow growth phenotypes have been reported for multiple transformed bacterial species, including extracellular and intracellular pathogens. Understanding the genes and pathways responsible for the slow growth phenotype provides the opportunity to develop attenuated vaccines as well as bacteriostatic antibiotics. Transformed *Anaplasma marginale*, a rickettsial pathogen, exhibits slow growth *in vitro* and *in vivo* as compared to the parent wild type strain, providing the opportunity to identify the underlying genes and pathways associated with this phenotype.

**Results:**

Whole genome transcriptional profiling allowed for identification of specific genes and pathways altered in transformed *A. marginale*. Genes found immediately upstream and downstream of the insertion site, including a four gene operon encoding key outer membrane proteins, were not differentially transcribed between wild type and transformed *A. marginale.* This lack of significant difference in transcription of flanking genes and the large size of the insert relative to the genome were consistent with a *trans* rather than a *cis* effect. Transcriptional profiling across the complete genome identified the most differentially transcribed genes, including an iron transporter, an RNA cleaving enzyme and several genes involved in translation. In order to confirm the trend seen in translation-related genes, K-means clustering and Gene Set Enrichment Analysis (GSEA) were applied. These algorithms allowed evaluation of the behavior of genes as groups that share transcriptional status or biological function. Clustering and GSEA confirmed the initial observations and found additional pathways altered in transformed *A. marginale*. Three pathways were significantly altered as compared to the wild type: translation, translation elongation, and purine biosynthesis.

**Conclusions:**

Identification of perturbed genes and networks through genome wide transcriptional profiling highlights the relevance of pathways such as nucleotide biosynthesis, translation, and translation elongation in the growth phenotype of obligate intracellular bacteria. These genes and pathways provide specific targets for development of slow growing attenuated vaccines and for bacteriostatic antibiotics.

## Background

The ability to genetically manipulate bacteria has been fundamentally important for both basic biological discovery and translational research to develop new vaccines and antibiotics. Experimental alteration of the genetic content of prokaryotic pathogens has revealed both expected functional relationships and unexpected phenotypic consequences. Slow growth phenotypes induced by transformation have been reported for both free living bacteria such as *Escherichia coli* and *Salmonella*[1,2], as well as for facultative intracellular bacteria including *Legionella* and *Listeria*[3,4]. Identifying the genes, pathways, and mechanisms that underlie slow growth phenotypes of transformed bacteria has the potential to provide new approaches for development of effective vaccines and antimicrobials. Bacteria that have a lower rate of replication *in vivo* can shift the race between onset of disease and development of an effective immune response in favor of the host, thus allowing development of attenuated vaccines that induce immunity without significant clinical disease. Correspondingly, several genetically altered pathogens have been tested as live vaccine candidates [5-7]. In addition, identification of genes and pathways underlying slow growth phenotypes can reveal targets for development of bacteriostatic antibiotics, drugs that slow replication to the point where the host responses can clear the infection.

Despite the intriguing possibilities afforded by the slow growth phenotype, very little is known about the alterations that characterize this trait, especially in obligate intracellular pathogens where transformation technology has lagged behind that of bacteria capable of growing in cell-free media [8]. We have recently observed this growth phenotype in transformed *Anaplasma marginale*, the type species of the genus *Anaplasma* and a member of the Order *Rickettsiales*. Using a mariner transposon approach, *A. marginale* was transformed and genes encoding TurboGFP and spectinomycin/streptomycin resistance were inserted [9]. The *in vitro* and *in vivo* infectivity and stability, including transmission through the natural tick vector of *A. marginale,* has been established for the mutant (hereafter referred to as AmTR) [10]. Importantly, similar to wild type *A. marginale*, AmTR established infection in cattle, was transmitted by a competent arthropod vector species, and persisted in immune competent animals. However, AmTR not only required longer subculture intervals in ISE6 cells *in vitro* but had a clear slow growth phenotype *in vivo* as shown by the following observations as compared to wild-type *A. marginale*: i): calves infected with AmTR had longer prepatent periods prior to detectable bacteremia; ii) peak bacteremia levels were ten-fold lower; and iii) the bacterial levels in tick salivary glands were of 100-fold lower (10^3.41±0.25^ versus 10^5.40±0.54^) [10].

In this study, *A. marginale* is used as a representative model of an obligate intracellular pathogen to describe the genetic and transcriptional changes that characterize the slow growth phenotype. As size [11,12], genomic position [13] and codon usage [14] of an insert have been shown to affect the growth phenotype, these were analyzed for AmTR. Next, RNA-seq was used to identify transcriptional modifications that characterize the slow growth phenotype. For this, the transcriptional profiles of wild-type and AmTR were examined, providing a comparative steady-state snapshot of specific mRNA abundance. This comparison included both local and global analysis. For the former, the transcriptional status of genes flanking the insertion site in AmTR was analyzed. For the latter, whole genome transcriptional profiling was used to identify genes that were significantly differentially transcribed. Finally, clustering methods and Gene Set Enrichment Analysis were used to evaluate the behavior of groups of genes and pathways. Herein we report the results of these analyses and present the results in the context of the mechanisms that underlie the slow growth phenotype.

## Methods

### Insert analysis

The OPTIMIZER web server was used to perform predictions and adjustments in codon usage for the insert present in AmTR [15]. The ‘one amino acid–one codon’ method, a guided random method based on a Monte Carlo algorithm, was used. The *A. marginale* str. St. Maries codon usage table was extracted from Artemis [16].

### Cell culture and RNA-seq

The accession number for this RNA-seq study is: SRP014580. Three T75 flasks of ISE6 cells were infected with either wild type *A. marginale* or AmTR, representing three biological replicates. Both inoculums were originally derived from the St. Maries strain of *A. marginale*. The cell lines were maintained in ISE6 cells cultured at 34°C as previously described [9,17,18]. When passage 27 of either wild type or AmTR in a T75 cell culture flask infected greater than 80% of ISE6 cells, as determined by examination of Giemsa stained cytospin preparations, cell cultures were mechanically lysed by passaging 50 times through a bent 27 g needle attached to a 6 mL luer lock syringe, then filtered through a 2 μm syringe filter (Whatman) which allows passage of *A. marginale* but not cellular debris. The filtrate was pelleted by centrifugation, and then washed in PBS. The final pellet was resuspended in 200 μL PBS before adding 1 mL of TRIzol (Invitrogen). Total RNA was isolated from wild type or AmTR-infected ISE6 cell culture using TRIzol (Invitrogen), per manufacturer directions. Eukaryotic sequences were negatively selected through hybridization using the MICROBEnrich kit (Ambion). The Duplex‒Specific thermostable Nuclease (DSN) normalization protocol was applied to all samples. Samples were sequenced with Illumina technology with 100 bp reads, each sample was run on 1/3 of a lane. Data was processed using CLC Genomics Workbench (CLC Bio). Mapping parameters were adjusted to map a maximum number of reads to the reference bacterial genomes. The distribution of the expression values for all samples was analyzed and compared. Normalization by quantiles was applied to adjust the distributions for further comparison. Fold changes with respect to RPKM values were calculated [19]. Two different tests were applied to evaluate the statistical significance of fold changes: Kal and Baggerly’s statistical tests on proportions [20,21].

### Comparative transcriptional analysis

Expression was measured using quantitative reverse transcription PCR (qPCR) using the SYBR Green ER RT-PCR Kit (Invitrogen). Briefly, 200 ng of RNA were processed with the SuperScript® VILO™ cDNA Synthesis Kit (Invitrogen) to obtain cDNA. The steady state, single copy gene *msp*5 was used to calibrate the qPCR. Relative expression ratios were calculated by a mathematical model, which includes efficiency correction of individual transcripts through the REST software [22] with modifications as previously described [23]. This procedure was used for verification of transcriptional differences found by RNA-seq. The variance of the normalized expression values of the genes flanking the insert was evaluated through ANOVA paired with an F-test.

### K-means clustering

We used K-means clustering to assign the mean transcriptional value of each gene to the cluster whose center is nearest. Lloyd’s algorithm was used for these experiments [24]. Euclidean distance was used as distance metric; five partitions were used to generate the clusters. For each gene, the mean gene expression value over all input samples was subtracted. Normalized expression values were used for clustering.

### Gene Set enrichment analysis

The Gene Set Enrichment Analysis (GSEA) test described by Tian et al. [25] was used. Briefly, the test implicitly calculates and uses ANOVA statistic for multiple group experiments for each feature, as measures of association. For each category, the test statistics for the features in that category are summed and a category based test statistic is calculated as this sum divided by the square root of the number of features in the category. In order to annotate the *A. marginale* genome with Gene Ontology we used the Comprehensive Microbial Resource [26]. Briefly, we matched feature IDs in the *A. marginale* genome to synonyms and gene product form IDs from the Comprehensive Microbial Resource. Additionally, we complemented this source with manual curation of the annotation file, including genes reported in the Kyoto Encyclopedia of Gene and Genomes (KEGG) [27].

## Results/discussion

### Insert characteristics

The nature of the construct used for transformation can be a source for phenotypic alterations. The effect of insert size on bacterial fitness (measured by growth rate), has been previously shown for bacteria with intermediate genome sizes [11,12]. Inserts that ranged from 2.9 to 12.6 kb were used to transform either *E. coli* or *Bacillus subtilis*. In both studies, bacteria transformed using the largest inserts displayed slower growth rates. These larger inserts represent a small proportion of the chromosome they are inserted in: 0.2 and 0.3% of the total genome size in *E. coli* and *B. subitlis,* respectively. The insert used for transformation of *A. marginale* represents 0.4% of its single chromosome. Obligate intracellular pathogens in the *Rickettsia* and *Coxiella* genera have been transformed with inserts that vary in size, ranging from 0.001 to 0.5% of their chromosomes [28-30]. As these transformants displayed different growth phenotypes, an association between insert size and slow growth seems unlikely.

Foreign genes and their unfamiliar codon usage can affect bacteria through slow translation efficiency, difficultly folding the foreign protein, and protein degradation [31,32]. The issue of codon usage is highlighted for bacteria with unusual genomic G + C content, such as the obligate intracellular pathogens in the order *Rickettsiales*[33,34]. In order to analyze the relationship of the insert codon usage with that of *A. marginale*, we used OPTIMIZER, a web server that optimizes a DNA sequence using pre-computed codon usage tables [15]. Comparison of the insert with the recipient *A. marginale* revealed a Codon Adaptation Index (CAI) of 0.802. CAI values range from 0, when the codon usage of a sequence and that of the reference set are very different, to 1 when both codon usages are the same. These results suggest that codon usage is an unlikely explanation for reduced growth rate in AmTR.

### Whole genome transcriptional profiling

RNA-seq was used to compare whole genome transcriptional profiles of wild type and AmTR. This allowed for identification of individual genes whose transcriptional status segregates with the phenotype of interest. As obligate intracellular pathogens are dependent on their eukaryotic host cells, RNA samples are significantly contaminated by host transcripts. Illumina technology and selective hybridization allowed us to obtain sufficient depth to compare the transcriptomes of wild type and AmTR (Table 1). The number of reads mapped to the *A. marginale* genome ranged from 1,667,455 to 9,895,940, with AmTR providing fewer reads than wild type despite being harvested from cultures with similar levels of infection.

**Table 1:** **Reads mapped to the*A. marginale*St. Maries genome**

**Replicate**	**Total reads**	***A. marginale*reads**	**% of reads mapped to*A. marginale***	**Average matched read length**
**AmTR1**	59,342,223	1,667,455	2.8	100
**AmTR2**	54,170,723	1,816,895	3.4	100
**AmTR3**	76,734,051	2,474,678	3.2	100
**WT1**	62,196,873	8,494,596	13.7	100
**WT2**	65,911,848	8,220,811	12.5	100
**WT3**	103,604,396	9,895,940	9.5	100

AmTR: transformed, slow growth phenotype *A. marginale.*

WT: wild type *A. marginale.*

As genomic location has been identified as a source for phenotypic variation in transformed bacteria, the initial focus was on genes flanking the insert [13]. Integration of the 4.5-kb insert in AmTR occurred between the putative transcriptional regulator, *tr* and *ndk* at nucleotide position 1026363. The insertion site is immediately upstream to the major surface protein 2 (*msp2*) operon; the operon encodes four outer membrane proteins, OpAG1-3 and MSP2 [35]. Although modulation of the outer membrane has been shown to affect bacterial metabolism and growth [36], comparison of normalized expression values (RPKM) of the operon transcripts between the AmTR and the wild type (Table 2) through ANOVA paired with an F-test showed no significant difference (p = 0.99). We extended the analysis to an additional 14 genes flanking the insert; there was also no significant difference between AmTR and the wild type (p = 0.94).

**Table 2:** Transcriptional status of genes flanking the insertion site of AmTR

	Normalized RPKM values*	
**Feature ID**	**AmTR**	**WT**
***czcD***	291	2079
***ccmB***	374	245
***dksA***	379	779
***dtbS***	570	57
***ftsY***	400	191
***icd***	256	843
***recJ***	185	55
***AM1135***	61	11
***pcnB***	1407	185
***ndk***	2803	1119
***AM1138***	2878	3841
***omp1***	2523	1375
***opag3***	1073	193
***opag2***	317	531
***opag1***	385	346
***msp2***	239	1170
***AM1145***	177	1357
***AM1146***	157	413
***AM1148***	120	112
***orfX***	55	45

*AmTR: transformed; *A. marginale* WT: wild type *A. marginale.*

**Figure 1:**
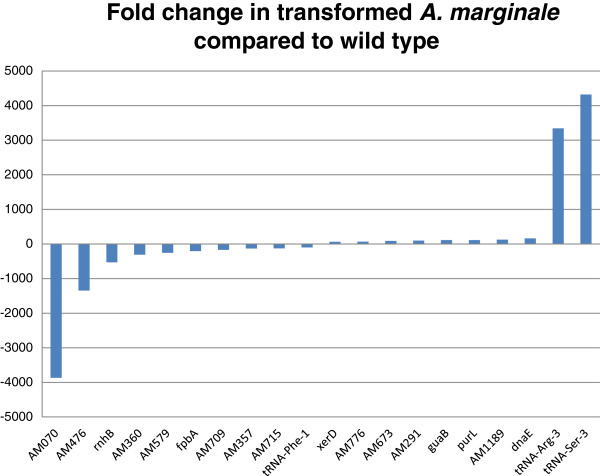
**Fold change in AmTR compared to wild type.** Locus tags for all genes are given on the X axis. The top 20 differentially transcribed genes identified across two replicates and two statistical tests and their fold changes are shown. All fold changes were found to be statistically significant at p < 5.05E-5 across both statistical tests.

Since the genes immediately adjacent to the insertion site were not significantly altered, we next identified the most differentially transcribed genes across all replicates. The top ten down and up regulated genes are shown in Figure 1. These genes were all significantly differentially transcribed (p < 1E-05) (Additional file 1 & Figure 1). Four of the top differentially transcribed genes (*AM360*, *AM357*, *rnhB* and *fpba*), one of the genes flanking the insertion site (*ndk*) and a gene involved in translation (*rpmE*) were evaluated with qPCR to validate the RNA-seq data (Additional file 2).

The RNA-seq analysis revealed genes that were significantly differentially transcribed, including genes with predicted functions, such as *rnhB, fpba*, *tRNA-Phe-1*, *xerD, guaB, purL*, dnaE, *tRNA-Arg-3* and *tRNA-Ser-3*. Gene *rnhB*, which encodes RNase H, an enzyme that specifically cleaves the RNA strand of RNA/DNA hybrids and plays a role in removing RNA primers from Okazaki fragments [37], was significantly down regulated in AmTR. Although it is known that this gene is dispensable in several bacterial models, knockout mutants of *rnhB* show growth rates that are about half of that of the wild type [38]. Another gene with predicted function that was found to be significantly down regulated in AmTR was *fpba,* which encodes an iron binding protein. The most common strategy of iron acquisition in bacteria is the production of high-affinity ferric iron binding molecules that can sequester iron from the host’s iron-binding proteins. Knock-out of iron acquisition mechanisms has been shown to attenuate pathogenic bacteria and impair their growth [39,40]. One example of this can be found in *Streptococcus pyogenes,* where inactivation of an iron transporter affected growth *in vivo*[41]. The transcriptional status of *fpba* and *rnhB*, together with evidence for reduced growth in other bacterial pathogens with impaired iron transport systems or RNase H enzymes, is consistent with the phenotype exhibited by AmTR.

### Differentially transcribed groups of genes and pathways

**Figure 2:**
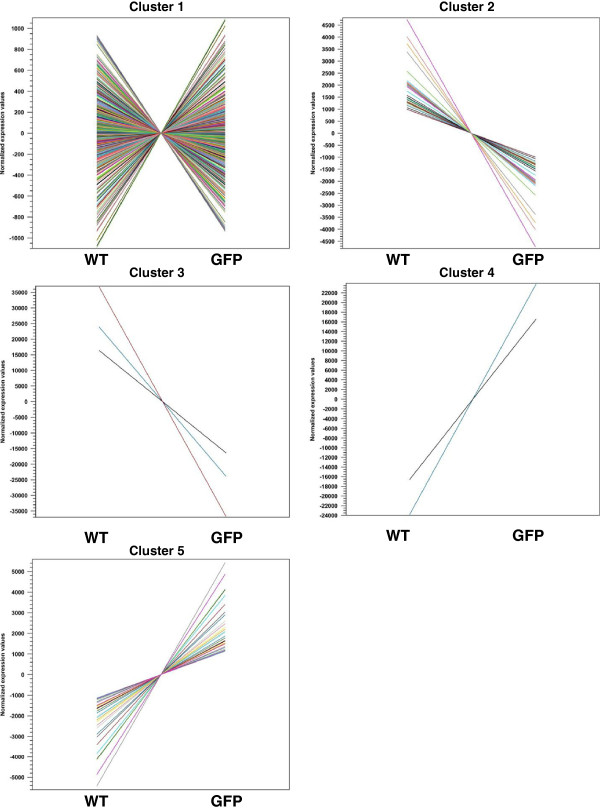
**Clusters identified through k-means clustering of AmTR versus wild type.** The 5 clusters identified in this study are displayed. The Y axis shows normalized expression values (RPKM). The X axis displays the values found for wild type (WT) and AmTR*.* Each colored line represents a different gene included in the cluster. The mean gene expression value over all input samples was subtracted to each gene’s value. Cluster affiliation for each gene can be found in Additional file 1.

Most phenotypes are the result of more complex genetic changes than divergence at a single gene or locus, or even a few loci [42]. Evaluation of the transcriptional status of groups of genes revealed that genes involved in translation exhibited a trend: of 61 genes, 50 were found to be significantly down regulated in AmTR (Additional file 3)*.* A first step toward addressing the challenge of statistically evaluating genes as groups is the use of clustering techniques. We used K-means clustering in order to validate a trend we were able to identify through observation and find further relationships. Most genes (882) fell into Cluster 1, which is comprised of genes that were either slightly up or down regulated between wild type and AmTR (Figure 2 & Additional file 1). The second cluster included 34 genes that were clearly down regulated in AmTR. Twenty of these genes are involved in translation and translation elongation. Cluster 3 included three genes that were down regulated in AmTR. The difference in average expression values for these genes was much greater than the differences seen in Cluster 2. This cluster included an additional two genes involved in translation. Cluster 4 incorporated two genes that were significantly up regulated in AmTR: *AM291* and *guaB*. Finally, Cluster 5 included 31 genes that were significantly up regulated in AmTR. This cluster included genes involved in translation as well as nucleotide biosynthetic pathways: *carB*, *purL* and *pyrG*.

Clusters 2 and 3 were of particular interest as they identified genes that were down regulated in AmTR compared to wild type. A large proportion (45%) of the genes involved in translation and translation elongation were included in these two clusters, validating our observation. Interestingly, it has been previously shown that mutations and transcriptional alterations in genes involved in translation provoke slow growth of *E. coli*[43], and three of these, *rpoB*, *rpsL* and *rpsE,* were also found to be altered in AmTR.

Although the effect on genes involved in translation seems clear, the main difficulty in analysis lies not in the identification of differentially expressed genes but in the interpretation of how these may interact. The problem is compounded when genes within a given pathway have only moderate changes in transcription that are not captured by the examination of only the most highly regulated genes. This appears to hold true for the translation related genes evaluated in this study. Although a trend was clear in this pathway, genes with small differences in transcriptional activity did not cluster with the most differentially regulated ones. In order to accurately identify perturbed pathways the technique used should consider the distribution of pathway genes in the entire list of genes as well as adjust for the correlation structure [44,45]. Consequently, we applied GSEA to our whole genome transcriptional profiling data. This analysis grouped over 500 genes into 165 different Gene Ontology categories. Eleven pathways were significantly affected transcriptionally (p < 0.05) (Additional file 4 & Table 3). Three specific pathways, translation, translation elongation, and purine biosynthesis were the most impacted (p < 0.001) (Table 3). GSEA confirmed our observations and identified additional pathways that were affected in AmTR. It is not surprising to find that both translation and translation elongation pathways were altered, as it has been shown that reduced translation-transcription coupling impacts the overall transcription and translation elongation rate [46].

**Table 3:** Significantly affected pathways in wild type vs. AmTR comparison through GSEA

**GO Category**	**Description**	Lower tail*	Upper tail*
**6412**	Translation (PMID:16482227 [ISS] Pfam:PF00573)	0.0044	0.9956
**6414**	Translational elongation (PMID:16482227 [ISS] Pfam:PF00889)	0.0071	0.9929
**15940**	Pantothenate biosynthetic process (PMID:12721629 [ISS] TIGR_TIGRFAMS:TIGR00018)	0.0158	0.9842
**6434**	Seryl-tRNA aminoacylation (PMID:16482227 [ISS] TIGR_TIGRFAMS:TIGR00414)	0.0322	0.9678
**15986**	ATP synthesis coupled proton transport (PMID:16482227 [ISS] TIGR_TIGRFAMS:TIGR01216)	0.0343	0.966
**8654**	Phospholipid biosynthetic process (PMID:12721629 [ISS] Swiss-Prot:O31752)	0.0343	0.9657
**19363**	Pyridine nucleotide biosynthetic process (PMID:16482227 [ISS] TIGR_TIGRFAMS:TIGR00550)	0.0381	0.9619
**6265**	DNA topological change (PMID:16482227 [ISS] TIGR_TIGRFAMS:TIGR01051)	0.9648	0.0352
**6364**	rRNA processing (GO_REF:0000012 [ISS] Swiss-Prot:P21513)	0.9673	0.0327
**917**	Barrier septum formation (PMID:16482227 [ISS] TIGR_TIGRFAMS:TIGR00172)	0.9899	0.0101
**9152**	Purine ribonucleotide biosynthetic process (PMID:16482227 [ISS] TIGR_TIGRFAMS:TIGR00184)	0.9997	0.0003

*Lower/Upper tail: The different pathways evaluated in through GSEA are shown. Lower and Upper tail values show the mass in the permutation based p-value distribution below or above the value of the test statistic.

Additionally GSEA allowed for identification of other significantly altered pathways such as purine biosynthesis. This is consistent with the finding that two genes involved in the purine biosynthesis pathway are among the top differentially transcribed genes: *guaB* and *purL*. Several previous reports have described the importance of nucleotide biosynthesis in bacteria. For instance, certain auxotroph mutants of *Salmonella*[47], *Staphylococcus aureus*[48] and *Steptococcus pneumoniae*[49] have shown impaired growth. These studies suggest that purines and selected amino acids are scarce in different environments and essential to pathogen growth. Impairment in nucleotide biosynthesis would further underlie the slow growth phenotype.

## Conclusions

Understanding the basis for the slow growth phenotypes of transformed bacteria provides targets for development of attenuated vaccines and bacteriostatic antibiotics. For the *Anaplasma marginale* transformant, AmTR, the slow growth phenotype is specifically relevant as it is manifest both in the mammalian host and the arthropod vector, expanding the opportunities for blocking transmission and disease. The AmTR slow growth phenotype results from a *trans* effect of the insertion and is consistent and is likely to be independent from the impact of a relatively large insert:genome ratio. Genome-wide transcriptional analysis identified individual genes such as *fpba* and *rnhB*, which were transcribed at significantly lower levels in AmTR as compared to wild type and encode functions consistent with reduced bacterial growth. The application of Gene Set Enrichment Analysis (GSEA) and clustering analysis to the large data sets generated in this study revealed significant transcriptional alterations in three pathways: translation, translation elongation and purine biosynthesis. Identification of these perturbed networks highlights the relevance of sequential relatively minor changes in individual genes of a pathway, which collectively have a pronounced phenotypic effect. This understanding moves beyond a one gene-one phenotype model and can accelerate translation of discovery into improved infection control.

## Abbreviations

AmTR: Transformed *A. marginale*; RPKM: Reads Per Kilobase per Million mapped reads; GSEA: Genes Set Enrichment Analysis; KEGG: Kyoto Encyclopedia of Genes and Genomes; CAI: Codon Adaptation Index

## Competing interests

The authors declare that they have no competing interests.

## Authors’ contributions

SAP, GKH, GHP, KAB conceived the experiments; SAP, GKH performed the experiments; SAP, GKH, KAB analyzed the data; SAP, GKH, GHP, KAB wrote and edited the manuscript. All authors read and approved the final manuscript.

## Supplementary Material

Additional file 1 **Whole genome transcriptional profiling of AmTR versus wild type*A. marginale.*** The genome wide transcriptional comparison of wild type and AmTR across three biological replicates is reported. Fold changes, total gene reads, raw and normalized expression values, p-values for Baggerly’s test and Cluster affiliation for each feature are shown. Click here for file

Additional file 2 **Validation of the transcriptional status of 6 genes through qPCR.** The fold changes of 6 genes found through RNA-seq (blue) and qPCR (red), across two biological replicates are reported. Click here for file

Additional file 3 **Fold change found for genes involved in translation pathway.** The fold changes found for genes involved in translation pathway in AmTR compared to wild type are reported. P values smaller than 1E-16 are reported as 0. Click here for file

Additional file 4 **GSEA on whole genome transcriptional profiling of wild type*A. marginale*** and AmTR. The different pathways evaluated in through GSEA are shown. Lower and Upper tail values show the mass in the permutation based p-value distribution below or above the value of the test statistic. Click here for file
