# Supplementary figures and images for: Transcriptional pathways associated with the slow growth phenotype of transformed Anaplasma marginale
Source: BMC Genomics. 2013 Apr 22;14:272. doi: 10.1186/1471-2164-14-272 (PMC3646689; doi:10.1186/1471-2164-14-272)

## Slide 1
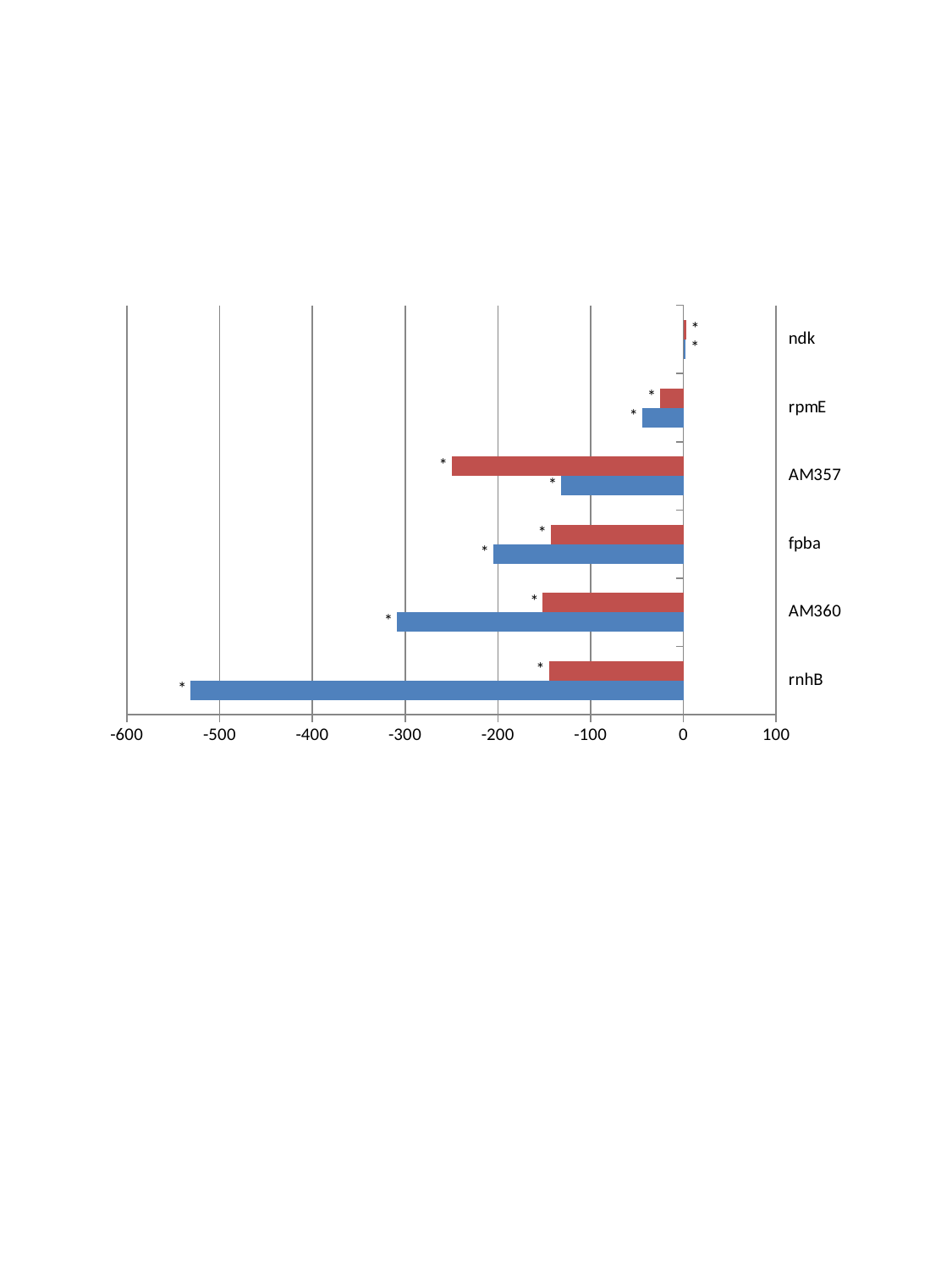

### Chart
| Category | | |
|---|---|---|
| rnhB | -531.1311079564679 | -144.92 |
| AM360 | -308.7448818053419 | -151.51 |
| fpba | -205.13 | -142.85 |
| AM357 | -131.65568522195576 | -249.547 |
| rpmE | -44.48916285294699 | -24.937 |
| ndk | 2.503259000435799 | 2.8 |

Supplement: Additional file 2 — Validation of the transcriptional status of 6 genes through qPCR. The fold changes of 6 genes found through RNA-seq (blue) and qPCR (red), across two biological replicates are reported. [file 1471-2164-14-272-S2.pptx]
