# Supplementary material for: Transcriptional pathways associated with the slow growth phenotype of transformed Anaplasma marginale
Source: BMC Genomics. 2013 Apr 22;14:272. doi: 10.1186/1471-2164-14-272 (PMC3646689; doi:10.1186/1471-2164-14-272)
Supplement: Additional file 3 — Fold change found for genes involved in translation pathway. The fold changes found for genes involved in translation pathway in AmTR compared to wild type are reported. P values smaller than 1E-16 are reported as 0. [file 1471-2164-14-272-S3.docx]

**Additional file 3: Fold change found for genes involved in translation pathway**

| Feature ID | **Fold Change in transformed *A. marginale* compared to wild type** | **Kal's Z-test P-value** |
| --- | --- | --- |
| rpmE | -44.5 | 0* |
| rplL | -33.4 | 4E-15 |
| rpiB | -15.5 | 0 |
| rpmG | -12.2 | 0 |
| rplA | -11.0 | 0 |
| rplJ | -9.9 | 1E-14 |
| rpsT | -8.7 | 0 |
| rplY | -6.7 | 8E-15 |
| rpoH | -6.3 | 1E-14 |
| rplS | -6.1 | 0 |
| rpmH | -5.9 | 0 |
| rplP | -5.6 | 0 |
| rplK | -5.0 | 0 |
| rpmF | -4.9 | 0 |
| rpmJ | -4.9 | 0 |
| rplU | -4.7 | 0 |
| rpmC | -4.5 | 1E-14 |
| rpsM | -3.2 | 0 |
| rpmB | -3.2 | 0 |
| rpmA | -3.1 | 0 |
| rpsD | -3.0 | 6E-15 |
| rpoD | -3.0 | 0 |
| rplF | -2.7 | 0 |
| rpsB | -2.6 | 1E-14 |
| rpsC | -2.5 | 0 |
| rpsP | -2.4 | 2E-14 |
| rplM | -2.4 | 0 |
| rpoZ | -2.4 | 2E-15 |
| rpsN | -2.3 | 0 |
| rpsH | -2.3 | 0 |
| rpsU | -2.3 | 3E-15 |
| rpe | -2.3 | 1E-07 |
| rplR | -2.2 | 0 |
| rpmI | -2.2 | 4E-15 |
| rplE | -2.2 | 0 |
| rpsF | -2.0 | 1E-14 |
| rpsL | -1.9 | 1E-14 |
| rplX | -1.7 | 4E-09 |
| rplV | -1.7 | 0 |
| rplO | -1.7 | 1E-12 |
| rpsQ | -1.7 | 2E-08 |
| rplN | -1.7 | 2E-07 |
| rpsI | -1.5 | 7E-08 |
| rpsS | -1.5 | 1E-10 |
| rpsE | -1.5 | 1E-06 |
| rplC | -1.4 | 2E-10 |
| rpsA | -1.3 | 2E-07 |
| rplB | -1.3 | 0.0016 |
| rpsR | -1.2 | 0.0438 |
| rplD | -1.1 | 0.6961 |
| rpsJ | -1.0 | 0.0728 |
| rplI | 1.1 | 0.0002 |
| rplT | 1.1 | 1E-14 |
| rplW | 1.2 | 5E-09 |
| rpsG | 1.2 | 1E-15 |
| rpoA | 1.4 | 0 |
| rpsO | 1.4 | 0 |
| rpsK | 1.5 | 0 |
| rplQ | 2.1 | 2E-14 |
| rpoC | 4.9 | 0 |
| rpoB | 8.7 | 0 |

*P values smaller than 1E-16 are reported as 0
